# The One-Way Linear Effect, a first order optical effect

**DOI:** 10.1016/j.heliyon.2023.e19590

**Published:** 2023-09-01

**Authors:** Gianfranco Spavieri, Espen Gaarder Haug

**Affiliations:** a Centro de Física Fundamental, Universidad de Los Andes, Mérida, 5101 Venezuela; b Norwegian University of Life Sciences, Ås, Norway

**Keywords:** Light propagation, Sagnac effect, One-way speed of light, Relative simultaneity, Lorentz invariance

## Abstract

In the One-Way Linear Effect, the round-trip time interval $T_{0}^{\prime }$ taken by light to propagate around a moving linear closed contour, is measured by a device. If the contour changes velocity by Δ*v*, the round-trip interval $T^{\prime }(X^{\prime },\Delta v)$ turns out to depend on the device position $X^{\prime }$ and Δ*v*. The variation $\Delta T^{\prime }=T^{\prime }(X^{\prime },\Delta v)-T_{0}^{\prime }$, related to Δv/c, is experimentally observable by means of standard interferometry, ring laser techniques, or high precision time-delay detectors of light pulses. Being sensitive to velocity variations, if experimentally confirmed the One-Way Linear Effect may have relevant applications in inertial guidance systems and related areas. Furthermore, the One-Way Linear Effect can be used to confirm Lorentz invariance by testing relative simultaneity *versus* absolute simultaneity.

## 1 Introduction

**Figure 1:**
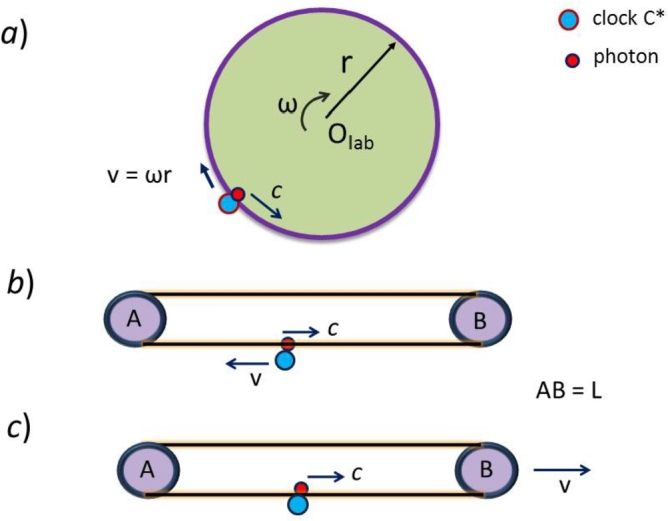
*a*) In the circular Sagnac effect, two counter-propagating photons (only a single photon is shown) are emitted from the device C* and travel on the circumference of the rotating platform. C* measures the difference Δ*T* of the photons' arrival times after a round trip. *b*) In the linear Sagnac effect, device C* is moving with velocity *v* relative to the stationary frame AB and emits counter-propagating photons traveling in an optical fiber that slides frictionless around pulley A and B. *c*) In the reciprocal Sagnac effect, the frame AB of the optical fiber moves with velocity *v* relative to the stationary device C* that emits photons counter-propagating along the optical fiber.

Among the relevant optical experiments historically related to light speed invariance, let's mention the following. The Michelson-Morley experiment [1] of 1887 provided a surprising null result that gave support to the theory of special relativity (SR) proposed by Einstein in 1905 [2] assuming light speed invariance and the Lorentz transformations (LT). The more sensitive Michelson-Gale experiment of 1925 [3], performed with an interferometer fixed on Earth, provided a non-null result. The standard Sagnac experiment [4], shown in Fig. 1-a, was carried out in 1913. The linear Sagnac experiment, shown in Fig. 1-b, was performed by Wang et al. [5], [6] in 2003. The linear Sagnac effect is considered to be equivalent to the standard Sagnac effect. In the Sagnac effects the emitter-receiver C* (clock or interferometer) is moving relative to a fixed contour where two light signals are counter-propagating and C* measures the difference $\Delta T=T_{⟸}-T_{⟹}$ of their round-trip times $T_{⟸}$ and $T_{⟹}$.

Recently, Spavieri and Haug [7] have proposed the Reciprocal Linear Sagnac Effect, shown in Fig. 1-c, where the measuring device C* is stationary and the contour is in relative motion. In the reciprocal effect, the observable Δ*T* is foreseen to be the same as in the linear Sagnac effect. There are, however, observable features of the linear Sagnac effect that differ from those of the reciprocal effect [7].

Following Post [8], for the Sagnac effects the difference Δ*T* is given by,

(1) $$\Delta T=T_{⟸}-T_{⟹}=\frac{2\pi r}{\gamma (c-v)}-\frac{2\pi r}{\gamma (c+v)}=\frac{4\gamma v\pi r}{c^{2}}=\frac{2\gamma vP}{c^{2}}=\frac{4\;\omega \cdot A}{c^{2}}\;\Delta T=T_{⟸}-T_{⟹}=\frac{2L}{\gamma (c-v)}-\frac{2L}{\gamma (c+v)}=\frac{4\gamma vL}{c^{2}}=\frac{2\gamma vP}{c^{2}},$$

where $T_{⟸}$ and $T_{⟹}$ represent the round-trip time of the co- and counter-moving light signals (or photons) along the contour of perimeter P=2πr in the standard (or circular) Sagnac effect and P=2L in the linear effect respectively. For the circular Sagnac effect, with v=ωr, the result (1) is usually expressed [8] in terms of the angular velocity *ω* and the area **A** enclosed by the light path.

Result (1) can be applied to the Michelson-Gale experiment, usually interpreted [9], [10] as a circular Sagnac effect, where the emitter-receiver device (or clock C*) is fixed on the frame of the interferometer where light propagates, as in the case of the Michelson-Morley experiment. However, in the Michelson-Gale experiment, the light path has the form of a rectangle of considerable area and, being placed on the surface of the rotating Earth, it rotates relative to the Earth Centered Inertial (ECI) frame. Hence, being analogous to the circular Sagnac effect, the Michelson-Gale experiment can measure the angular velocity $\omega_{E}$ of the Earth [10]. At present, experiments based on the Sagnac effect using ring laser techniques are performed routinely on Earth [11], [12] to measure $\omega_{E}$ with a precision $\delta \omega /\omega_{E}<10^{-8}$. Therefore, the Michelson-Gale experiment can be considered to be equivalent to a circular Sagnac experiment and its non-null result is due to the fact that the system composed of C* and the rectangular light path fixed on the Earth's surface is rotating relative to the ECI frame, where the speed of light is assumed to be *c*.

In the Michelson-Gale and the equivalent circular Sagnac experiment, the velocity *v* of C* is uniform and the two experiments can detect velocity variations due to rotation. It is thus feasible that there is an experimental set up, here denoted as One-Way Linear Effect based on the LT and light speed invariance, capable of detecting linear velocity variations. The purpose of our Letter is to show how the One-Way Linear Effect can be theoretically verified. A realistic test of the One-Way Linear Effect, possible with present technology, is described.

The Sagnac effect finds important applications in inertial guidance systems extremely sensitive to rotations, such as ring laser gyroscope and other optical systems. It is premature to indicate the applications that the One-Way Linear Effect can have. Nevertheless, what is important is that the techniques involved in this type of effects are very sensitive. Then, as well as in the case of the reciprocal linear Sagnac effect ([7]), potential uses of the One-Way Linear Effect might be the detection of movements of seismic origin (and corresponding direction) or velocity variations of aircraft due to air turbulence.

## 2 Round-trip time interval of a particle moving along a linear contour

The circular and linear Sagnac effects are usually described in the inertial frame of reference of the stationary contour, where the speed of light is assumed to be *c*. Interpretations of the Sagnac effects in the frame comoving with device C* are given by several authors [13], [14], [15], [16], [17], [18], [19], [20], [21], [22], [23], [24], [25], [26], [27], [28].

In the linear Sagnac effect of Fig. 1-b the arm AB of the contour is stationary and clock C* is moving clockwise with uniform speed *v* along the contour, going from the lower to the upper section of the contour and vice versa. While sliding around the pulley of radius *R* during the short time *η*, the device C* changes direction of motion at the pulley A (or B). Locally, the speed *v* of C* relative to the stationary contour is always constant. In the linear experiment by Wang et al. [5], device C* stays on the lower contour section in uniform rectilinear motion during the round-trip time $T≃T_{⟸}≃T_{⟹}$ and therefore C* does not turn around the pulleys A or B. Nevertheless, since the relative speed *v* between C* and contour is constant, the theoretical prediction (1) for $T_{⟸}$, $T_{⟹}$, and Δ*T* is maintained, even when C* turns around the pulley during the round-trip time *T*.

In the reciprocal linear Sagnac effect of Fig. 1-c, the device C* is stationary and the contour is moving relative to C*. For this effect, as long as C* is always on the lower (or upper) track of the contour during the round-trip interval *T*, the predictions for Δ*T* are the same as those of Fig. 1-b. However, the one-way predictions for $T_{⟸}$ and $T_{⟹}$ are different when C* is first on the lower and then on the upper track during the interval *T* ([7]).

Previous works [18], [19], [20] discuss the role of simultaneity in special relativity in the context of the circular and linear Sagnac effects and other special cases [21]. In these previous works, the device C* is moving relative to the contour where light propagates, but in the present One-Way Linear Effect the device C* is always fixed to and co-moving with the contour, while the contour velocity may be varying relative to an inertial frame. We show in Section 3 that, if the velocity of the contour changes by Δ*v*, the relevant feature of the One-Way Linear Effect foreseen by the LT is that the one-way round-trip time interval $T^{\prime }$ measured by C* depends not only on Δ*v*, but also on the position $X^{\prime }$ of C* on the contour, so that: $T^{\prime }=T^{\prime }(X^{\prime },\Delta v)$. This special feature is not present in the physical effects discussed in previous works [18], [19], [20], [21], [22] or other works related to the interpretation of the Sagnac effect.

A significant characteristic of the One-Way Linear Effect is that it represents an optical effect of the Sagnac type capable of detecting velocity variations. If its existence is confirmed experimentally, the One-Way Linear Effect may likely find important useful technological applications in inertial guidance systems and related areas, as has been the case for the Sagnac effect.

Another important aspect of the One-Way Linear Effect is that its special feature is foreseen by the LT based on relative simultaneity. Yet, as shown in the Appendix, this special feature is not predicted by theories based on absolute simultaneity. This fact suggests that, by exploiting the One-Way Linear Effect, it is possible in principle to test relative simultaneity *versus* absolute simultaneity. Tests discriminating relative from absolute simultaneity are uncommon in literature, because in the context of relativistic theories it is generally assumed that synchronization of spatially separated clocks is arbitrary and relative and absolute simultaneity are not observable [29]. However, in our effect, the measuring device is only the single clock C*, which does not require synchronization. Thus, the experiment on the One-Way Linear Effect described in Section 3 appears feasible for testing Lorentz invariance. Being capable of discriminating relative from absolute simultaneity, the One-Way Linear Effect represents a definite advance for the comprehension of the role of simultaneity in relativistic theories.

In the One-Way Linear Effect experiment described in the next section we focus on the one-way prediction for $T^{\prime }$, as foreseen by the LT.

### 2.1 Contour in uniform motion

**Figure 2:**
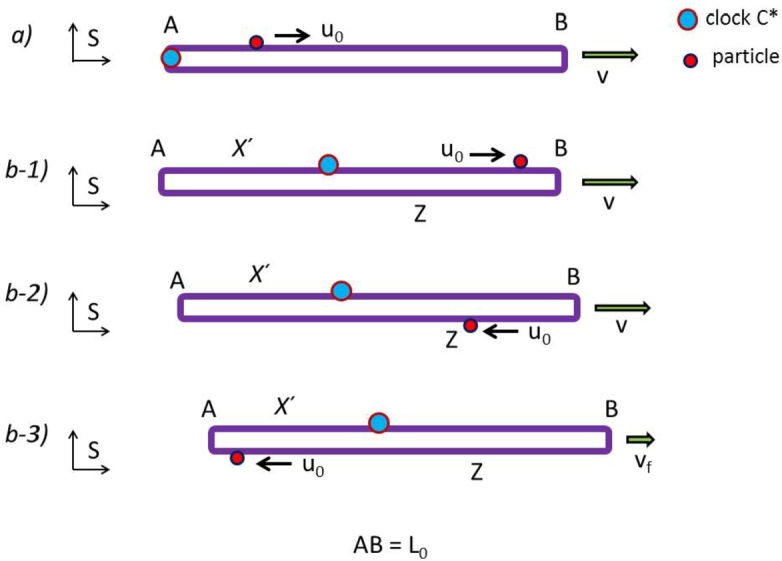
*a)* The contour AB is moving at speed *v* relative to frame *S*. After being emitted by C* at A, the particle moves always at the local speed *u*_0_ relative to the contour. When reaching point B, the particle changes direction and returns to C*, while the contour keeps moving at speed *v*. *b-1)* C*, placed on the contour upper section at the distance *X*′ from A, emits a particle that, traveling at the local speed *u*_0_, reaches B. *b-2)* The particle changes direction at B and moves on the lower section toward point Z. *b-3)* When the particle has covered the distance *L*_0_ from C* and passes by Z, the contour changes speed from *v* to *v*_*f*_. When reaching A, the particle changes direction and returns to C*.

Let us consider the linear contour of Fig. 2-a co-moving with frame $S^{\prime }$ at speed *v* relative to the lab frame *S*. The device C* is fixed to the contour at the end point A. A particle is moving on the contour upper section from A to B in the interval $t_{out}$ in the out trip, and back on the lower section from B to A in the interval $t_{ret}$ in the return trip, traveling at the local speed $u^{\prime }=u_{0}$ relative to the contour rest length AB $=L_{0}$. If the contour consists of an optical fiber of refractive index *n*, the particle can be a photon traveling at speed $u_{0}=c/n$. The results derived are valid also for the case, to be discussed elsewhere, of a curved contour forming a circular arc AB moving along the circumference.

In agreement with the relativistic velocity transformations, the particle speed in frame *S* is $u_{out}=(u_{0}+v)/(1+u_{0}v/c^{2})$ in the out trip and $u_{ret}=(u_{0}-v)/(1-u_{0}v/c^{2})$ in the return trip. With,

$$u_{out}-v=\frac{u_{0}}{\gamma^{2}(1+u_{0}v/c^{2})}\;u_{ret}+v=\frac{u_{0}}{\gamma^{2}(1-u_{0}v/c^{2})},$$

the resulting time intervals are,

(2) $$t_{out}=\frac{L_{0}}{\gamma (u_{out}-v)}=\frac{\gamma L_{0}(1+u_{0}v/c^{2})}{u_{0}}\to \frac{\gamma L_{0}(1+v/c)}{c}\;\;t_{ret}=\frac{L_{0}}{\gamma (u_{ret}+v)}=\frac{\gamma L_{0}(1-u_{0}v/c^{2})}{u_{0}}\;\to \frac{\gamma L_{0}(1-v/c)}{c}\;T=t_{out}+t_{ret}=\frac{2\gamma L_{0}}{u_{0}}\to \frac{2\gamma L_{0}}{c}\;T^{\prime }=\tau^{\prime }=t_{out}^{\prime }+t_{ret}^{\prime }=\frac{T}{\gamma }=\frac{2L_{0}}{u_{0}}\to \frac{2L_{0}}{c},$$

where in (2) $T^{\prime }=\tau^{\prime }$ represents the round-trip proper time interval measured by C*. The expressions to the right in (2) after the arrow → represent the results in free space when $u_{0}=c$.

Since C* is fixed to and co-moving with the contour, the round-trip interval *T* calculated from frame *S* is the same whether the particle travels one way clockwise or anti-clockwise along the contour starting from C* and returning to C*. Results (2) are independent of the position of C* relative to A, as long as the contour is in uniform motion during the interval *T*.

## 3 Contour in variable motion

If the velocity of the contour changes by Δ*v*, the interval $T^{\prime }=T^{\prime }(X^{\prime },\Delta v)$ depends on the initial distance $X^{\prime }$ of C* from point A and on Δ*v*.

The contour of rest length $2L_{0}$ shown in Figs. 2-b is in motion relative to the inertial frame *S*. A particle, emitted from C* located at the distance $X^{\prime }$ from A, travels (Fig. 2-b1) along the contour at the local speed $u_{0}$ and reaches point B where is reflected. When the particle has covered the distance $L_{0}$ from C*, it reaches point Z (Fig. 2-b2), being the distance of Z from B: ZB $=X^{\prime }$. At this moment the contour velocity is slowed by Δ*v* to $v_{f}=v-\Delta v$ in the negligible interval *η* (Fig. 2-b3) and the particle travels from Z to A, where it is reflected, and completes its round trip by reaching C* in the time interval *T*.

For simplicity's sake, let's consider the ideal case where *η* is negligible in comparison to *T* and, relative to $S^{\prime }$, the particle travels the short finite distance $\eta u_{0}\ll 2vL_{0}/c$ during the interval *η*. With this assumption, we may omit taking into account the details of the process when the contour changes velocity.

The particle is moving along the contour performing what we define as a one-way clockwise round trip starting from C*. A two-way trip consists of a clockwise trip from C* and back to C*, plus a counter-clockwise trip from C* and back to C*.

In the trip from C* to Z, the contour has uniform speed *v*. According to the LT, relative to frame *S* the speed of the particle is $u^{+}=(u_{0}+v)/(1+u_{0}v/c^{2})$ while traveling from C* to B. The time interval $t_{1}$ from C* to B is obtained from the equation $u^{+}t=(L_{0}-X^{\prime })/\gamma +vt$. While traveling from B to Z the particle speed is $u^{-}=(u_{0}-v)/(1-u_{0}v/c^{2})$ and the time interval $t_{2}$ from B to Z is obtained from the equation $X^{\prime }/\gamma -u^{-}t=vt$. With

$$u^{+}-v=\frac{u_{0}}{\gamma^{2}(1+u_{0}v/c^{2})}\;u^{-}+v=\frac{u_{0}}{\gamma^{2}(1-u_{0}v/c^{2})},$$

we find,

(3) $$t_{1}=\frac{L_{0}-X^{\prime }}{\gamma (u^{+}-v)}=\frac{\gamma (L_{0}-X^{\prime })(1+u_{0}v/c^{2})}{u_{0}}\to \frac{\gamma (L_{0}-X^{\prime })}{c}+\frac{\gamma v(L_{0}-X^{\prime })}{c^{2}}\;t_{2}=\frac{X^{\prime }}{\gamma (u^{-}+v)}=\frac{\gamma X^{\prime }(1-u_{0}v/c^{2})}{u_{0}}\to \frac{\gamma X^{\prime }}{c}-\frac{\gamma vX^{\prime }}{c^{2}}\;t_{1}+t_{2}=\frac{\gamma L_{0}}{u_{0}}+\frac{\gamma vL_{0}}{c^{2}}-\frac{2\gamma vX^{\prime }}{c^{2}}\to \frac{\gamma L_{0}}{c}+\frac{\gamma vL_{0}}{c^{2}}-\frac{2\gamma vX^{\prime }}{c^{2}}$$

Since in the short interval *η* the contour has decelerated to speed $v_{f}=v-\Delta v$, from Z to A the return speed of the particle is $u_{f}^{-}=(u_{0}-v_{f})/(1-u_{0}v_{f}/c^{2})$ and the corresponding time interval $t_{3}$ is obtained from the equation $(L_{0}-X^{\prime })/\gamma_{f}-u_{f}^{-}t=v_{f}t$. For the trip from A to C* the speed is, $u_{f}^{+}=(u_{0}+v_{f})/(1+u_{0}v_{f}/c^{2})$ and for the corresponding interval $t_{4}$ the equation is $u_{f}^{+}t=X^{\prime }/\gamma_{f}+v_{f}t$. With,

$$u_{f}^{-}+v_{f}=\frac{u_{0}}{\gamma^{2}(1-u_{0}v_{f}/c^{2})}\;u_{f}^{+}-v_{f}=\frac{u_{0}}{\gamma^{2}(1+u_{0}v_{f}/c^{2})},$$

we find,

(4) $$t_{3}=\frac{L_{0}-X^{\prime }}{\gamma_{f}(u_{f}^{-}+v_{f})}=\frac{\gamma_{f}(L_{0}-X^{\prime })(1-u_{0}v_{f}/c^{2})}{u_{0}}\to \frac{\gamma_{f}(L_{0}-X^{\prime })}{c}-\frac{\gamma_{f}v(L_{0}-X^{\prime })}{c^{2}}\;t_{4}=\frac{X^{\prime }}{\gamma_{f}(u_{f}^{+}-v_{f})}=\frac{\gamma_{f}X^{\prime }(1+u_{0}v_{f}/c^{2})}{u_{0}}\to \frac{\gamma_{f}X^{\prime }}{c}+\frac{\gamma_{f}vX^{\prime }}{c^{2}}\;t_{3}+t_{4}=\frac{\gamma_{f}L_{0}}{u_{0}}-\frac{\gamma v_{f}L_{0}}{c^{2}}+\frac{2\gamma v_{f}X^{\prime }}{c^{2}}$$

The round-trip proper time interval $T^{\prime }=$ $\tau^{\prime }$ measured by C* can be derived from the time transformation of the LT, which is $t^{\prime }=\gamma (t-vx/c^{2})$ when the contour speed is *v* relative to *S*. In the interval Δ*t* clock C* moves by Δx=vΔt and the proper time interval is $\Delta \tau^{\prime }=\Delta t^{\prime }=\gamma \Delta t(1-v^{2}/c^{2})=$ Δt/γ. Alternatively, we may use the inverse transformation, $t=\gamma (t^{\prime }+vx^{\prime }/c^{2})$ and consider the variation, $\Delta t=\gamma (\Delta t^{\prime }+v\Delta x^{\prime }/c^{2})$. Since $\Delta t^{\prime }=$ $\Delta \tau^{\prime }$ is measured by C* at the same position ($\Delta x^{\prime }=0$), we find again, $\Delta \tau^{\prime }=\Delta t^{\prime }=\Delta t/\gamma$ (i.e., $\tau^{\prime }=T/\gamma$). After the time interval $\Delta t=t_{1}+t_{2}$, when the particle has reached Z, the contour and the device C* change velocity. Then, for the successive time interval $\Delta t_{f}=t_{3}+t_{4}$, C* is moving at speed $v_{f}$ and now, $\Delta t_{f}^{\prime }=\Delta t_{f}/\gamma_{f}$. Hence, with $\Delta v=v-v_{f}$ relations (3) and (4) give,

(5) $$T^{\prime }(X^{\prime },\Delta v)=\Delta t^{\prime }+\Delta t_{f}^{\prime }=\frac{L_{0}}{u_{0}}+\frac{vL_{0}}{c^{2}}-\frac{2vX^{\prime }}{c^{2}}+\frac{L_{0}}{u_{0}}-\frac{v_{f}L_{0}}{c^{2}}+\frac{2v_{f}X^{\prime }}{c^{2}}=\frac{2L_{0}}{u_{0}}+\frac{\Delta v}{c}\frac{L_{0}}{c}-\frac{2\Delta v}{c}\frac{X^{\prime }}{c}\to \frac{2L_{0}}{c}+\frac{\Delta v}{c}\frac{L_{0}}{c}-\frac{2\Delta v}{c}\frac{X^{\prime }}{c}.$$

We can see from (5) that, besides depending on $X^{\prime }$, the interval $T^{\prime }(X^{\prime },\Delta v)$ depends on Δ*v* and is sensitive to velocity variations of the first order in Δv/c.

There is a remarkable difference between $T^{\prime }$ in (2) and $T^{\prime }(X^{\prime },\Delta v)$ in (5). As seen from an observer co-moving with the contour when in uniform motion, result (2) indicates that the particle, traveling at the local speed $u_{0}$, in the interval $T^{\prime }$ covers the ground distance $2L_{0}$ as expected. However, if the contour changes velocity by Δ*v*, result $T^{\prime }(X^{\prime },\Delta v)$ in (5) indicates that the particle covers the different ground distance $2L_{0}+2u_{0}\Delta v(L_{0}-2X^{\prime })/c^{2}\to 2L_{0}+2\Delta v(L_{0}-2X^{\prime })/c$.

Results (5) have been derived in the inertial frame *S* where the speed of light is assumed to be *c* and using the velocity transformations provided by the LT. The same results (5) can be derived from the inertial frame $S^{\prime }$, $S^{\prime \prime }$, or any other inertial frame taking into account the effect due to relative simultaneity, as considered in the Appendix.

**Testing the one-way effect**

In the case considered above, the round-trip interval $T^{\prime }=T_{⟸}^{\prime }$ for a given velocity variation Δ*v*, refers to a one-way (clockwise) co-moving particle traveling along the contour. In the Sagnac effect the interval $T_{⟹}^{\prime }$ for a counter-moving particle differs from $T_{⟸}^{\prime }$ and $\Delta T^{\prime }=T_{⟸}^{\prime }-T_{⟹}^{\prime }\neq 0$. In our case, however, since C* is fixed on the contour, the interval $T_{⟹}^{\prime }(X^{\prime },\Delta v)$ for a counter-moving particle is the same as in (5) for the co-moving particle. Then, for the usual case of counter-propagating particles we have the null result, $\Delta T^{\prime }=T_{⟸}^{\prime }(X^{\prime },\Delta v)-T_{⟹}^{\prime }(X^{\prime },\Delta v)=0$ and, in our case, the dependence on $X^{\prime }$ and Δ*v* of the one-way interval $T_{⟸}^{\prime }(X^{\prime },\Delta v)$ cannot be revealed.

However, the one-way round-trip interval $T^{\prime }(X^{\prime },\Delta v)$ can be compared with the round-trip time interval $T_{0}^{\prime }=2L_{0}/u_{0}$ taken by a particle to propagate along an optical fiber of length $2L_{0}$ forming a coil that can be placed at the position of C* and co-moving with it. Let $d_{0}$ represent the dimension of the coil where the spiraling particle moves back and forth. According to (2), for uniform motion the time interval corresponding to each turn in the coil is $\tau_{partial}^{\prime }=2d_{0}/u_{0}$, independent of velocity. Therefore, during the time interval when the coil is moving at the uniform speed *v* relative to *S*, by adding up the partial intervals $\tau_{partial}^{\prime }$ until the length $L_{0}$ is covered, the expected proper time interval is $T_{0}^{\prime }/2=L_{0}/u_{0}$. Again, during the time interval when the coil is moving at the uniform speed $v_{f}$ relative to *S*, we have $T_{0}^{\prime }/2=L_{0}/u_{0}$. Then, the total length $2L_{0}$ of the coil is covered after the round-trip proper time interval $T_{0}^{\prime }=2L_{0}/u_{0}$, which is the same as in the case of a stationary coil. Hence, the device C* can measure the non-null difference,

(6) $$\Delta T^{\prime }(X^{\prime },\Delta v)=T^{\prime }(X^{\prime },\Delta v)-T_{0}^{\prime }=\frac{\Delta vL_{0}}{c^{2}}-\frac{2\Delta vX^{\prime }}{c^{2}}.$$

**Figure 3:**
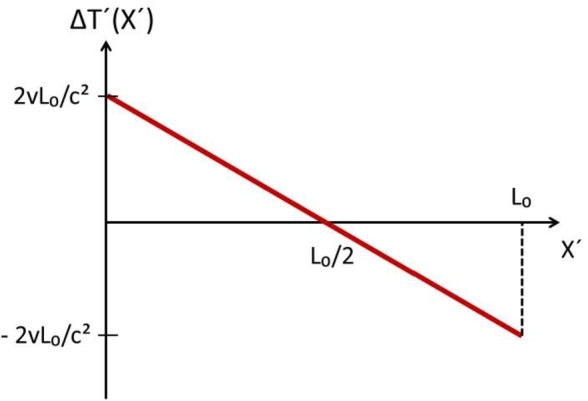
Special case Δ*v* = 2*v*. The difference $\Delta T^{\prime }(X^{\prime })=T^{\prime }(X^{\prime })-T_{0}^{\prime }$ as a function of the position of device C* along the contour. The maximum variation occurs at *X* = 0 and *X* = *L*_0_. When C* is at *x*′ = *L*_0_/2, the round-trip interval is $T^{\prime }(X^{\prime })=T_{0}^{\prime }=2L_{0}/c$ and Δ*T*′(*L*_0_/2)=0.

The plot of $\Delta T^{\prime }(X^{\prime },\Delta v)$ as a function of the position $X^{\prime }$ of C* is given in Fig. 3, where we assume that the contour changes direction of motion with velocity variation Δv=2v.

For the one-way effect described above, we have made some assumptions in order to simplify calculations, such as that the interval *η* is negligible and the velocity change occurs when the particle covers the distance $L_{0}$. However, in principle the calculations can be performed in general and, thus, we expect to have $T^{\prime }=T^{\prime }(X^{\prime },\Delta v,\eta ,L,...)$ depending on the various parameters that can affect the result.

**Expected precision of the measuring device.**

For testing the existence of the optical effect it is sufficient to perform, for example, measurements at $X^{\prime }=0$ and $X^{\prime }=L_{0}/2$. The measurable difference $\Delta T^{\prime }=\Delta T^{\prime }(X^{\prime },\Delta v)$ in (6) represents the prediction of standard special relativity based on the LT. $\Delta T^{\prime }$ may be measured with standard interferometry or by means of ring laser techniques [8], [30], [31]. With standard interferometry there could be some problems of stability because of the change of speed of the contour. Alternatively, detectors that act as clocks may be used to measure the time of flight of light pulses. For detectors capable of measuring $\Delta T^{\prime }$, there are techniques that may resolve femtosecond ($10^{-15}(s)$) [32] or even attosecond ($10^{-18}(s)$) [33], [34] pulses of laser light. The value of $\Delta T^{\prime }$ is comparable to that of the standard quantities measured in the Sagnac effects, which are within the range of the sensitivity of existing detectors. The main challenge for this type of experiment is probably the one related to the mechanical difficulties involving the motion of the contour, which requires speed variations for performing the test.

## 4 Conclusions

**Figure 4:**
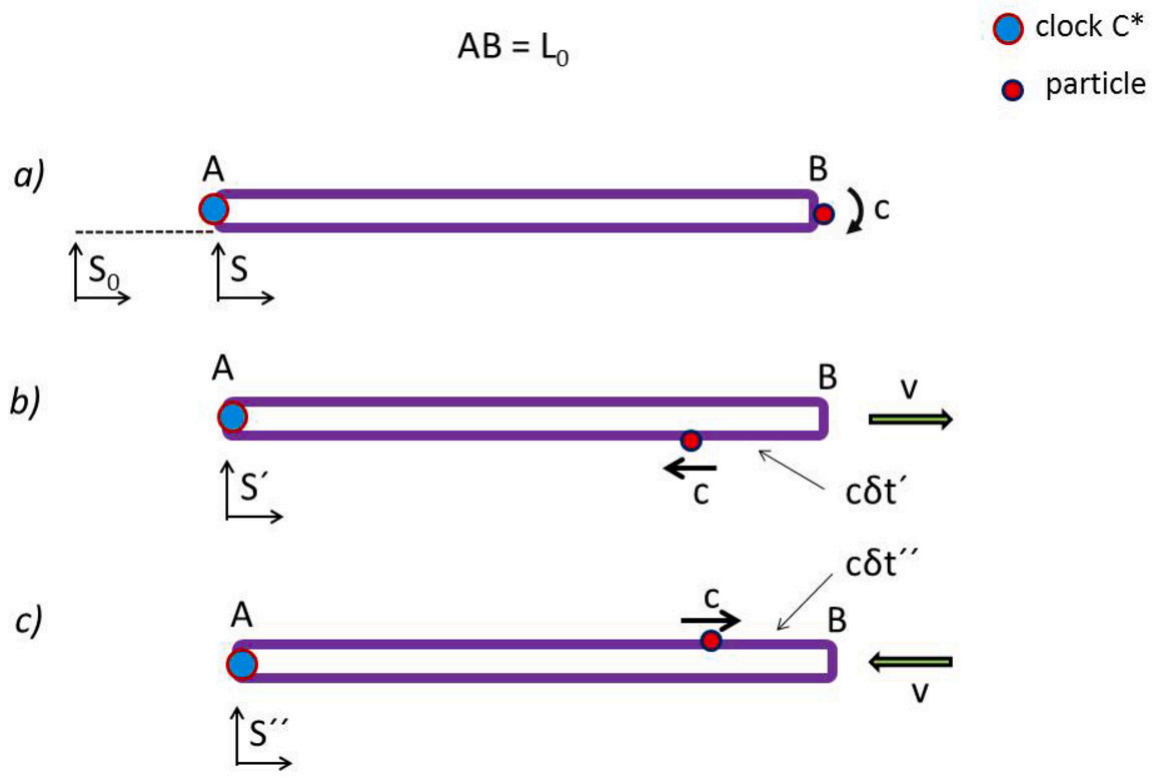
Effect of relative simultaneity between frames in relative motion. At *t* = *t*′ = *t*″ = 0 the origins of frames *S*, *S*′, and *S*″ coincide at A. *a*) As seen from frame *S*, at the moment (*t* = 0) the contour is changing direction of motion, the particle (a photon) is at point B. *b*) As seen from frame *S*′ moving at the relative speed +*v*, at *t*′ = 0 the photon is already on the lower section at the distance *cδt*′ from B. *c*) As seen from frame *S*″ moving at the relative speed −*v*, at *t*″ = 0 the photon is still on the upper section at the distance *cδt*″ from B.

We have shown that, for our One-Way Linear Effect, when the contour changes velocity, the LT foresee the $X^{\prime },\Delta v$-dependent round-trip time interval $T^{\prime }(X^{\prime },\Delta v)$ for the particle, or light, propagation. When $T^{\prime }(X^{\prime },\Delta v)$ is compared with the round-trip interval $T_{0}^{\prime }=2L_{0}/c$ corresponding to a stationary contour, we find that the LT foresee the observable non-null variation $\Delta T^{\prime }(X^{\prime },\Delta v)=T^{\prime }(X^{\prime },\Delta v)-T_{0}^{\prime }$ in (6). The difference $\Delta T^{\prime }(X^{\prime })$ is plotted in Fig. 4 for the special case Δv=2v and its maximum is $2vL_{0}/c^{2}$, experimentally observable with present technology.

As in the case of the Sagnac effect, which after more than a century has found important uses in the development of technology, it is feasible that, if experimentally confirmed, the One-Way Linear Effect can find similar important applications in inertial guidance systems or other related areas.

## CRediT authorship contribution statement

Gianfranco Spavieri and Espen Gaarder Haug: Conceived and designed the experiments; Performed the experiments; Analyzed and interpreted the data; Contributed reagents, materials, analysis tools or data; Wrote the paper.

## Declaration of Competing Interest

The authors declare that they have no known competing financial interests or personal relationships that could have appeared to influence the work reported in this paper.

## Data Availability

No data was used for the research described in the article.

## Appendix A

If we wish to derive results (5) from an inertial frame other than frame *S*, we have to take into account that, since simultaneity is not conserved with the LT, the contour ends A and B change direction of motion simultaneously in frame *S*, but not in the contour or other frames.

Within the assumption $\eta u_{0}\ll 2vL/c$ and, thus, neglecting the interval $\eta \ll T^{\prime }$ when the velocity change takes place, the contour frame is co-moving with the inertial frame $S^{\prime }$ before changing direction of motion and with the inertial frame $S^{\prime \prime }$ immediately after. In short, we may see how the mechanism of relative simultaneity works between frames in relative motion, as follows. Let us consider the case $X^{\prime }=0$ when the particle is emitted from C* at A and travels at speed *c*. Furthermore, let the contour change direction of motion with $v_{f}=-v$ and Δv=2v. In this case, from (5) the round trip interval is,

(7) $$T^{\prime }=T_{out}^{\prime }+T_{ret}^{\prime }=\frac{2L_{0}}{c}+\frac{2vL_{0}}{c^{2}},$$

which we interpret in terms of the out trip from A to B ($T_{out}^{\prime }=L_{0}/c+vL_{0}/c^{2}$) and the return trip from B to A ($T_{ret}^{\prime }=L_{0}/c+vL_{0}/c^{2}$).

For the out trip as seen from frame *S*, we consider the instant when the particle reaches point B (Fig. 4-a) and the contour has traveled the distance $vT_{out}$ from the original position $S_{0}$, shown in Fig. 4-a. Then, to keep it simple, we assume that the origins of *S*, $S^{\prime }$, and $S^{\prime \prime }$ coincide at point A at $t^{\prime \prime }=t^{\prime }=t=0$, as in Fig. 4.

According to the time transform of the LT, $t^{\prime }=\gamma (t-vx/c^{2})$, the event “particle at B with $x_{B}=L_{0}/\gamma$ at t=0” occurs in the past at $t_{B}^{\prime }=\gamma (0-vx_{B}/c^{2})=-vL_{0}/c^{2}=-\delta t^{\prime }$. Therefore, at the present time $t^{\prime }=0$ on $S^{\prime }$, after reflection at B at $t_{B}^{\prime }=-\delta t^{\prime }$ the particle has moved by $c\delta t^{\prime }$ toward the contour end point A (Fig. 4-b). Hence, as seen from $S^{\prime }$, the path length covered in the out trip is $L_{0}$ from A to B on the upper contour section, and $c\delta t^{\prime }$ from B toward A on the lower section in the interval $\delta t^{\prime }$, i.e., $L_{0}+c\delta t^{\prime }$. In agreement with the interpretation based on relative simultaneity, the corresponding measured time interval is, $T_{out}^{\prime }=L_{0}/c+\delta t^{\prime }=L_{0}/c+vL_{0}/c^{2}$.

On the return trip, the contour changes direction of motion and we have to consider the frame $S^{\prime \prime }$ co-moving with the contour just after changing direction of motion (Fig. 4-c). According to the time transform of the LT, $t^{\prime \prime }=\gamma (t+vx/c^{2})$, for $S^{\prime \prime }$ the event “particle at B with $x_{B}=L_{0}/\gamma$ at t=0” will occur in the future at $t_{B}^{\prime \prime }=\gamma (0+vx_{B}/c^{2})=vL_{0}/c^{2}=\delta t^{\prime \prime }$. Therefore, at the present time $t^{\prime \prime }=0$ on $S^{\prime \prime }$, the particle has not yet reached B and is on the upper contour section (Fig. 4-c), and will reach B after traveling the distance $c\delta t^{\prime \prime }$. Afterwards, traveling at speed *c* from B toward A, the particle covers the path length $L_{0}$ and reaches C* after traveling the distance $L_{0}+c\delta t^{\prime \prime }$, corresponding to the measured time interval $T_{ret}^{\prime }=L_{0}/c+\delta t^{\prime \prime }=L_{0}/c+vL_{0}/c^{2}$.

In conclusion, when the contour changes direction of motion, the round-trip interval measured by C* is not $2L_{0}/c$, but is given by (7) where, according to standard special relativity based on the LT, the additional contribution $2vL_{0}/c^{2}=\delta t^{\prime }+\delta t^{\prime \prime }$ comes from the effect of relative simultaneity between the frames in relative motion ($S^{\prime }$ and *S* in the out trip, and $S^{\prime \prime }$ and *S* in the return trip).

**Interpreting the optical effect in the context of theories assuming absolute simultaneity.**

In the interpretation of optical phenomena in the context of theories assuming absolute simultaneity, we are looking for the preferred frame where the light speed can be assumed to be *c*. For the Michelson-Morley experiment, the Sagnac effect, the reciprocal linear Sagnac effect, and other optical phenomena, the natural choice corresponds to the inertial frame where the contour is stationary, Maxwell's equations are valid, and the electromagnetic waves, constrained to move along the contour, propagate at speed *c*. Then, we may assume that the contour is carrying along in its motion the electromagnetic waves propagating at the local speed *c*, regardless of the contour state of motion. If simultaneity is conserved in this scenario, even with the contour changing velocity, $\delta t^{\prime }=\delta t^{\prime \prime }=0$ and there are no variations in the relative positions of the particle on the contour, as it occurs with relative simultaneity in Fig. 4-a, 4-b, and 4-c. Hence, by assuming absolute simultaneity we have $T^{\prime }=2L_{0}/c$ independent of $X^{\prime }$ and $\Delta T^{\prime }(X^{\prime })=0$ in (6), representing a null result, unlike the predictions of the LT based on relative simultaneity.

## References

[1] Michelson A.A., Morley E.W. (1887). On the relative motion of the Earth and the luminiferous ether. Am. J. Sci..

[2] Einstein A. (1905). Zur Elektrodynamik bewegter Körper. Ann. Phys..

[3] Michelson A., Gale H. (1925). The effect of the Earth's rotation on the velocity of light, II. Astrophys. J..

[4] Sagnac G. (1913). L'ether lumineux demontre par l'effet du vent relatif d'ether dans un interferometre en rotation uniforme. C. R. Acad. Sci..

[5] Wang R., Zhengb Y., Yaob A., Langley D. (2003). Modified Sagnac experiment for measuring travel-time difference between counter-propagating light beams in a uniformly moving fiber. Phys. Lett. A.

[6] Wang R., Zheng Y., Yao A. (2004). Generalized Sagnac effect. Phys. Rev. Lett..

[7] Spavieri G., Haug E.G. (2023). The reciprocal linear effect, a new optical effect of the Sagnac type. Open Phys..

[8] Post E.J. (1967). Sagnac effect. Rev. Mod. Phys..

[9] Anderson R., Vetharaniam I., Stedman G.E. (1998). Conventionality of synchronisation, gauge dependence and test theories of relativity. Phys. Rep..

[10] Silberstein L. (1921). The propagation of light in rotating systems. J. Opt. Soc. Am..

[11] Schreiber K.U., Gebauer A., Igel H., Wassermann J., Hurst R.B., Wells J.-P.R. (2014). From a tabletop experiment to the variation of the Earth's rotation. C. R. Phys..

[12] Stedman G.E. (1997). Ring-laser tests of fundamental physics and geophysics. Rep. Prog. Phys..

[13] Lee C. (2020). Simultaneity in cylindrical spacetime. Am. J. Phys..

[14] Klauber R.D. (1999). Comments regarding recent articles on relativistically rotating frames. Am. J. Phys..

[15] Klauber R.D. (2002). Anomalies in relativistic rotation. J. Sci. Explor..

[16] Selleri F. (1996). Noninvariant one-way velocity of light. Found. Phys..

[17] Selleri F. (1997). Noninvariant one-way speed of light and locally equivalent reference frames. Found. Phys. Lett..

[18] Spavieri G., Gillies G.T., Haug E.G., Sanchez A. (2019). Light propagation and local speed in the linear Sagnac effect. J. Mod. Opt..

[19] Spavieri G., Gillies G.T., Haug E.G. (2021). The Sagnac effect and the role of simultaneity in relativity theory. J. Mod. Opt..

[20] Spavieri G. (2012). On measuring the one-way speed of light. Eur. Phys. J. D.

[21] Spavieri G. (2021). Light propagation on a moving closed contour and the role of simultaneity in special relativity. Eur. J. Appl. Phys..

[22] Spavieri G., Haug E.G. (2022). Testing light speed invariance by measuring the one-way light speed on Earth. Phys. Open.

[23] Gift S.J.G. (2015). Analysis of recent attempts by Kassner to resolve Selleri's paradox. Appl. Phys. Res..

[24] Kipreos E.T., Balachandran R.S. (2016). An approach to directly probe simultaneity. Mod. Phys. Lett. A.

[25] Kipreos E.T., Balachandran R.S. (2021). Assessment of the relativistic rotational transformations. Mod. Phys. Lett. A.

[26] Lundberg R. (2019). Critique of the Einstein clock variable. Phys. Essays.

[27] Lundberg R. (2021). Travelling light. J. Mod. Opt..

[28] Field J.H. (2017). The Sagnac effect and transformations of relative velocities between inertial frames. Fund. J. Mod. Phys..

[29] Mansouri R., Sexl R.U. (1977). A test theory of special relativity: I. Simultaneity and clock synchronization. Gen. Relativ. Gravit..

[30] Malykin G.B. (2000). The Sagnac effect: correct and incorrect explanations. Phys. Usp..

[31] Malykin G.B. (2014). Sagnac effect in ring lasers and ring resonators. How does the refraction index of the optical medium influence the sensitivity to rotation?. Phys. Usp..

[32] Ludlow D.A., Boyd M.M., Ye J., Peik E., Schmidt P.O. (2015). Optical atomic clocks. Rev. Mod. Phys..

[33] Kim J., Chen J., Cox J., Kärtner F.X. (2007). Attosecond-resolution timing jitter characterization of free-running mode-locked lasers. Opt. Lett..

[34] Kwon D., Jeon C.G., Shin J., Heo M.S., Park S.E., Song Y., Kim J. (2017). Ultrafast, sub-nanometre-precision and multifunctional time-of-flight detection. Sci. Rep..

